# Mapping study on AI-based technologies in palliative care – a scoping study

**DOI:** 10.1186/s12904-025-01909-w

**Published:** 2025-10-28

**Authors:** Mariana Silva-Ferreira, Sara Cruz, Michael Sapateiro Luís, Maria Cândida Silva, Sara Monteiro-Reis, Rui Henrique, Carmen Jerónimo, Saint-Clair Lefèvre, Ambre Laplaud, Matthieu Frasca, Lucie Pollet, Lourdes Zurbanobeaskoetxea, Rosana Angles Barbastro, María Isabel Hidalgo García, Beatriz Jiménez Galán, Fátima González Palau, Diana Filipa Marques, Raquel Losada Durán

**Affiliations:** 1 Cancer Biology & Epigenetics Group, IPO Porto Research Center (CI-IPOP) Portuguese Oncology Institute of Porto (IPO Porto), R. Dr. António Bernardino de Almeida, Porto, 4200-072 Portugal; 2 Clinical Oncology Group, IPO Porto Research Center (CI-IPOP), Portuguese Oncology Institute of Porto (IPO Porto), R. Dr. António Bernardino de Almeida, Porto, 4200-072 Portugal; 3 CI-IPOP@RISE-Associate Laboratory and Porto Comprehensive Cancer Center Raquel Seruca (Porto.CCC), R. Dr. António Bernardino de Almeida, Porto, Portugal; 4 Doctoral Program in Nursing Sciences, ICBAS – School of Medicine and Biomedical Sciences –University of Porto, Rua Jorge Viterbo Ferreira 228, Porto, 4050-513 Portugal; 5 Oncology Nursing Research Unit, IPO Porto Research Center (CI-IPOP), Portuguese Oncology Institute of Porto (IPO Porto), R. Dr. António Bernardino de Almeida, Porto, 4200-072 Portugal; 6 Department of Surgical Oncology, Portuguese Oncology Institute of Porto (IPO Porto), R. Dr. António Bernardino de Almeida, Porto, 4200-072 Portugal; 7 Department of Palliative Care, Portuguese Oncology Institute of Porto (IPO Porto), R. Dr. António Bernardino de Almeida, Porto, 4200-072 Portugal; 8 Department of Pathology, Portuguese Oncology Institute of Porto (IPO Porto), R. Dr. António Bernardino de Almeida, Porto, 4200-072 Portugal; 9 Department of Pathology and Molecular Immunology, ICBAS – School of Medicine and Biomedical Sciences –University of Porto, Rua Jorge Viterbo Ferreira 228, Porto, 4050-513 Portugal; 10 Infinitivity Design Labs (IDL), Haute Loire, Laussanne, France; 11 Gérontopôle Nouvelle-Aquitaine, Limoges, France; 12 CHU de Bordeaux, Service d’accompagnement et de Médecine Palliative, Bordeaux, France; 13 Université de Bordeaux, Inserm U1219, Equipe EPIdémiologie du Cancer et des Expositions Environnementales EPICENE, Bordeaux, France; 14 Diputación Foral de Bizkaia, Regional Government of Bizkaia, Bilbao, Bizkaia Spain; 15 Servicio Aragonés de Salud, Hospital de Barbastro, Barbastro, Spain; 16 Consejería de Familia e Igualdad de Oportunidades, Gerencia de Servicios Sociales (GSS), Valladolid, Spain; 17 Instituto de Neurociencias y Bienestar, Universidad Siglo 21, Córdoba, Argentina; 18 Fundación INTRAS, Valladolid, Spain

**Keywords:** Palliative Care, Digital Health Technology, Artificial Intelligence, Patient-Centered Care, Scoping Review

## Abstract

**Background:**

The aging population and rising prevalence of chronic illnesses emphasize the importance of palliative care (PC), which focuses on enhancing patients' quality of life (QoL) while supporting their families and caregivers. PC integrates multidisciplinary interventions to alleviate the physical, psychological, social, and spiritual suffering of individuals facing serious or terminal illnesses. Concurrently, Artificial Intelligence (AI) advancements have been transforming the healthcare sector, particularly through Clinical Decision Support Systems (CDSS). Leveraged by advanced algorithms and machine learning (ML), these tools analyze large volumes of data to support diagnostics, personalized treatments, and early interventions. In PC, AI has demonstrated potential to enhance early diagnosis, identify support needs, and personalize end-of-life care. ML algorithms help predict symptoms and complications, enabling timely and effective interventions. However, challenges remain, including data privacy concerns, integration into clinical workflows, and ethical implications of AI in sensitive care contexts.

**Methods:**

We conducted a scoping review to map and analyze AI applications on PC. Articles published until May 2024 were identified in two electronic databases. From 542 records, 57 studies met the inclusion criteria. The review explored trends, benefits, and limitations of AI applications, highlighting tools for diagnostic and prognostic support, symptom tracking, shared decision-making, and communication with patients and families.

**Results:**

The findings highlight how digital technologies and AI are revolutionizing communication, care coordination, and symptom control in PC, unlocking remote care options. The review identified key advancements in symptom management, communication, decision support, telemedicine and education areas, while addressing barriers like ethical, legal, and accessibility concerns.

**Conclusions:**

By compiling evidence on AI use in PC, we aimed to empower professionals, researchers, and policymakers to promote more effective, ethical, and person-centered strategies. Ultimately, we provide insights for developing new technologies and establishing protocols that support the safe, equitable, and person-centered implementation of AI in palliative care, and highlight the need to prioritize early identification of patient needs, promote integration between hospital and community care, and establish protocols.

**Supplementary Information:**

The online version contains supplementary material available at 10.1186/s12904-025-01909-w.

## Background

The rising incidence of chronic illnesses and an aging population have underscored the critical role of palliative care (PC). Beyond symptom management and quality of life (QoL) enhancement, PC provides holistic support to patients, families, and caregivers, by addressing physical, psychological, social, and spiritual needs [1]. The demand for PC continues to grow worldwide, yet many patients still experience unmet needs due to fragmented services, late referrals, and limited resources. In this context, ensuring dignity, respect, and alignment with patients’ values and preferences is paramount.

Concurrently, advances in Artificial Intelligence (AI) are reshaping healthcare delivery. AI-powered Clinical Decision Support Systems (CDSS), driven by machine learning (ML) algorithms and natural language processing, analyze vast datasets to identify patterns and generate evidence-based recommendations [2, 3]. In various healthcare settings, AI has been employed for diagnostic assistance, risk prediction, symptom monitoring and personalized treatment recommendations [3]. These advancements have the potential to transform PC by enabling early identification of patient needs, improving care coordination and optimizing resource allocation [4, 5].

In the context of PC, AI applications are showing great promise in several key areas, being employed to facilitate communication, track and manage symptoms, predict mortality, detect psychological distress, and assess caregiver needs [6]. Predictive models, for instance, can help physicians anticipate disease progression, identify high-risk patients, and intervene proactively to prevent complications [7]. Additionally, the real-time monitoring of symptoms, such as pain or fatigue, allows for timely interventions [8]. In turn, chatbots and virtual assistants are emerging as valuable tools for patient communication and caregiver support, offering accessible guidance and emotional reassurance [9].

Despite the thriving progress, integrating AI into PC still presents notable challenges. Key barriers include the interpretability of algorithms, the risk of bias, and ethical concerns surrounding data privacy, informed consent, and transparency [3, 7, 10]. Cultural and organizational readiness, as well as interoperability with electronic health record (EHR) systems, also influence successful implementation [11, 12].

Furthermore, several knowledge gaps persist, and systematic evidence on the role of AI in PC is still scarce. Most studies are exploratory or limited to pilot implementations, with little evaluation of long-term outcomes or impact on person-centered care. Moreover, the influence of AI tools on the therapeutic relationship and ethical decision-making at the end of life remains largely unexplored. This gap highlights the need for comprehensive mapping of current knowledge to guide responsible integration.

Beyond technological advancements, the ethical and humanistic dimensions of AI in PC must be carefully considered. AI should complement – not replace – the compassionate and person-centered approach that defines PC. Ultimately, the goal is to ensure that AI-driven solutions uphold the dignity, autonomy, and comfort of patients while enhancing the quality and efficiency of PC delivery.

This scoping review aims to map the current landscape of AI applications in PC, identify areas of benefit, and highlight challenges that must be addressed to ensure that these technologies support, rather than diminish, the human touch in PC. By identifying knowledge gaps and opportunities for future research, this work may guide subsequent studies and inform evidence-based policies and practices.

## Methods

### Aims

This review aims to assess the use of AI technologies in PC, identifying advances, knowledge gaps and priority areas for future research. The current work seeks to explore how these tools may contribute to improving care, personalizing interventions and providing continuous support to patients and families, promoting a more integrated and humanized approach. Additionally, we also strive to identify barriers and facilitators related to ethical, legal, economic, and cultural aspects in order to provide valuable insights for researchers, healthcare professionals (HCPs), policymakers, and technology developers.

### Search strategy

Literature research was carried out using the PubMed and Scopus databases to trace the most relevant studies published from database inception to May 2024. The search strategy involved combining the terms “palliative care” and “technology”, with no limits or filters applied. The full electronic search queries for each database may be accessed in Additional File 1. Furthermore, reference lists of retrieved records were reviewed to identify additional studies of interest.

### Eligibility criteria

The citations of the articles retrieved were inserted in Rayyan, an online AI tool that allows remote collaboration for reviews’ screening. After removing duplicated files, the screening process involved two reviewers evaluating the titles and abstracts based on the following pre-established criteria: description, development, or implementation of any digital health technology aiming to enhance PC practices. As the main outcome of this review is to identify digital tools, platforms, or AI-based systems developed or applied to support the delivery, coordination, communication, decision-making, or personalization of PC delivery, articles that focused on the assessment of innovative treatments were excluded. Studies addressing ethical and legal dimensions were included, as long as they were connected to the development, implementation, or impact of AI in PC contexts. English was the mandatory language requirement for articles’ inclusion.

### Data extraction and synthesis

Full texts of the selected studies were thoughtfully read to assess eligibility, independently by the two reviewers. Thereafter, relevant information from the included studies was extracted using a standardized extraction template conceived by the team and categorized according to authorship, country, year of publication, disease of the target population, aim/general digital intervention purpose, type of technology used, user/operator or target parties, description of the technology concept, context of usage, advantages and limitations of the technology.

## Results

### Studies categorization

**Fig. 1:**
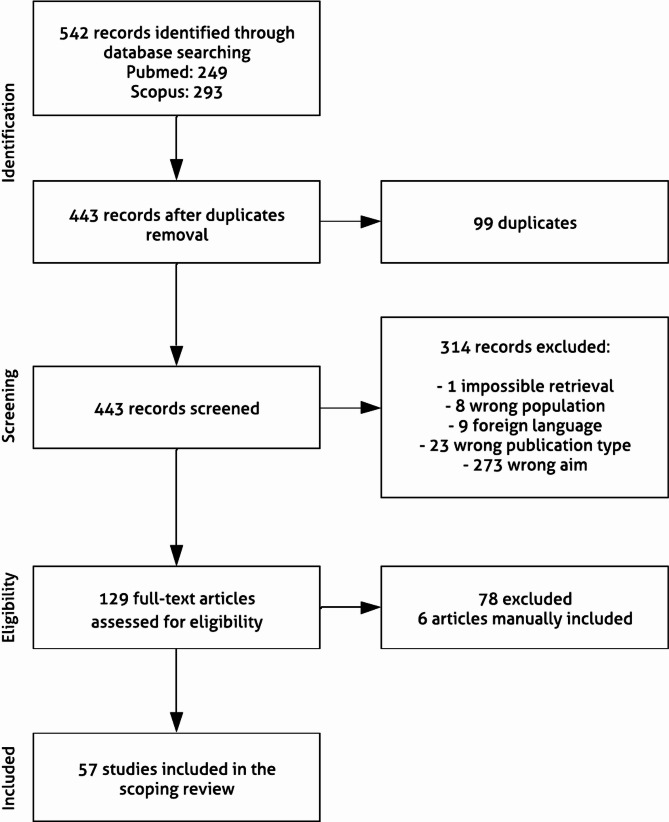
Flowchart of the literature selection process for the included studies. A total of 542 records were identified in two literature databases. After duplicates removal, 443 articles were screened, of which 129 full-texts were assessed. The final selection of included articles consisted of 57 studies

This scoping review provides a comprehensive understanding of digital health technology applications in PC delivery over the last 22 years, contemplating a total of 57 articles (Fig. 1).

Over time, the growing adoption of various types of digital technologies is evident. In fact, summarized in Table 1, it is possible to map the different applications of digital health technology implementation in healthcare and how it affects different clinical workflows. Particularly, the findings reveal that using AI in PC provision aims to support or improve symptoms control [13–30], communication [12, 21, 24, 25, 27, 29–45], advising and decision-making [7, 12, 18, 20, 23, 30, 33, 34, 39, 40, 42, 43, 45–48], telemedicine [13–17, 19–28, 31, 32, 34, 36, 44, 49–55], and education/training [23, 25, 34, 39, 46, 47, 53, 54, 56–59], which aligns with the classification of digital interventions, services, and applications in health established by the World Health Organization (WHO) [60]. Additionally, several studies have also assessed the effectiveness, usage patterns, and user perspectives on e-health technology [11–14, 16, 20, 22, 24, 27, 29, 35, 37, 39, 40, 43, 45, 53, 56–58, 61–66], while others designed protocols for new technologies’ implementation [19, 34, 42, 54, 59, 67].

**Table 1:** Summary of the 57 included articles and respective technologies. Overview of the included studies, indicating authors, year of publication, country, intervention category, name or type of technology and a brief description, to highlight key characteristics and thematic distribution across the literature

**Article**	**Year**	**Country**	**Category**	**Technology**	**Brief Description/Purpose**	**Ref.**
Coyle N., et al.	2002	USA	Telemedicine Communication	VideoPhone	Remote home care is supported by audio and video phones that operate on ordinary (analogue) telephone lines.	[31]
Saysell E., Routley C.	2003	UK	Counseling & Decision support Education	Teleconference	Teleconference for case discussions, advising and education among health teams.	[46]
Sugden S., et al.	2003	UK	Education Usefulness	CD-ROM	Interactive, educational CD-ROM on pain management in cancer.	[56]
Hebert M.A., et al.	2003	Canada	Telemedicine Communication	VideoPhone	Remote home care is supported by audio and video phones that operate on ordinary (analog) telephone lines.	[32]
Lind L., Karlsson D.	2004	Sweden	Telemedicine Symptoms control User's Experience	Digital Pen	Digital pens with built-in camera, image processor, and a communication unit that tracks, records and transmits handwriting as digital data, for pain management.	[13]
Bharadwaj P., et al.	2007	USA & India	Education Counseling	Teleconference	Teleconference for case discussions, advising and education among health teams.	[47]
Carr S.M., et al.	2008	UK	Education Counseling	Telephone advice line	Telephone line for case discussions, advising and education among health teams.	[33]
Lind L., et al.	2008	Sweden	Telemedicine Symptoms control User's Experience	Digital Pen	Digital pens with built-in camera, image processor, and a communication unit that tracks, records and transmits handwriting as digital data, for pain management.	[14]
Sudhamony S., et al.	2008	India	Telemedicine Communication Counseling & Decision support Education Implementation Protocol	Tele-Oncology network	ONCONET-Kerala is a telemedicine network system that integrates audio, video, and data conferencing. Enables tele-consultations, clinical discussions, and real-time interaction with patients. It also supports remote clinics and promotes education.	[34]
Bharucha A.J., et al.	2009	USA	Telemedicine Symptoms control	Several	Review of technologies with potential applications on dementia care, majorly in symptoms control and telemedicine.	[15]
El-Jawahri A., et al.	2010	USA	Decision support	Video	Video presenting the three levels of medical care to aid patients' decision-making for end-of-life care.	[48]
Cox A., et al.	2011	UK	Telemedicine Symptoms control User's Experience	Computerized assessment systems	HealthHUB™ and CareHUB™ are two systems of a computerized tool for patients and clinicians, respectively, to support monitoring and symptoms control.	[16]
Levy S., Steele G.	2012	Scotland	Telemedicine	Teleconference	Remote home care is supported by videoconferencing software using a computer.	[49]
Mouttham A., et al.	2012	Canada	Effectiveness	Computerized information system	PC information system (PAL-IS) is a solution proposal for interoperability challenges.	[11]
Chih M.Y., et al.	2013	USA	Telemedicine Symptoms control	Online assessment system	Online symptom reporting system to assess caregivers' preparedness and burden.	[17]
Stukenborg G.J., et al.	2014	USA	Symptoms control Decision support	Tablet + Online queries	Online questionnaire to collect patient-reported outcome measures, using a wireless touch screen tablet.	[18]
Thomas K., Moore G.	2015	Australia	Education Users' Experience	DVD	Educational DVD with informative videos to support caregivers.	[57]
van Gurp J., et al.	2015	Netherlands & USA	Telemedicine	Teleconference	Remote home care is supported by teleconsultations using a computer or a tablet.	[50]
Taylor A., et al.	2016	Australia	Telemedicine Symptoms control Counseling & Decision support Usefulness	Several	Remote home care is provided by videoconferencing using a tablet (iPad). Remote monitoring provided by a self-assessment app. Teleconference for case discussions, advising and education among health teams.	[20]
Guo Q., et al.	2017	Canada	Communication Users' Experience	Internet-based communication	"Keep in Touch (KIT)" - Internet-based communication provided by an iPad or computer for hospitalized patients to contact their families.	[35]
Klaas V.C., et al.	2017	Switzerland	Telemedicine Symptoms control Implementation Protocol	Wearable + App	Armband and smartphone with an associated app for distress and pain management.	[19]
Howard I.M., Kaufman M.S.	2018	USA	Telemedicine Symptoms control Communication	Several	Review on technologies with potential applications for patients with neuromuscular or musculoskeletal disorders, majorly in symptoms control, communication and telemedicine.	[21]
Calton B.A., et al.	2019	USA	Telemedicine	Several	Review on the main digital tools used for telemedicine in PC.	[51]
Jess M., et al.	2019	Denmark	Telemedicine	Teleconference	Systematic review on the use of video consultation in PC.	[52]
Nwosu A.C., et al.	2019	UK	Usefulness	Robots	SWOT analysis on the care support provided by robots.	[61]
Weck C.E., et al.	2019	Germany	Telemedicine Communication	Teleconference	Remote home care is supported by videoconferencing using a mobile telesystem app that allows to consult an expert.	[36]
Cruz-Oliver D.M., et al.	2020	USA	Education Usefulness	Video	Systematic review on educational videos for patients and caregivers.	[53]
Holland D.E., et al.	2020	USA	Telemedicine Education Implementation Protocol	Teleconference	Personalized care model with videos that provides guidance and advise for caregivers during patients' transition from the hospital to home.	[54]
Lai L., et al.	2020	USA	Education Usefulness	PalliCOVID	Progressive web application for clinicians with evidence-based clinical guidelines and educational resources.	[58]
Leniz J., et al.	2020	UK	Communication Usefulness	Electronic Palliative Care Co-ordination System (EPaCCS)	Systematic review on the state of science of EPaCCs - electronic registers that enables the documentation of updated information about patients.	[37]
Nguyen M., et al.	2020	Canada	Acceptance	Technology Acceptance Model (TAM)	TAM used as a theoretical framework to explore healthcare providers and administrators’ acceptance of digital technologies in PC.	[62]
Pavic M., et al.	2020	Switzerland	Telemedicine Symptoms control Usefulness	Wearable + App	Armband and smartphone with an associated app for distress and pain management.	[22]
Standing H., et al.	2020	UK	Communication Users' Experience	Electronic Palliative Care Co-ordination System (EPaCCS)	EPaCCs are electronic registers that enables the documentation of updated information about patients.	[12]
Disalvo D., et al.	2021	Australia	Telemedicine	Several	Systematic meta-review on virtual models that support PC delivery at home.	[55]
Dunleavy L., et al.	2021	UK	Communication	CovPall	Online multinational survey to map and understand specialist PC services innovations and practice changes in response to COVID-19.	[38]
Finucane A.M., et al.	2021	UK	Telemedicine Symptom control Decision support Education	Several	Systematic meta-review on digital health interventions applied in PC.	[23]
Nkhoma K.B., et al.	2021	UK	Communication Counseling Education Users' Experience	Several	Qualitative study on digital technologies used in PC to guide its development.	[39]
Oelschlägel L., et al.	2021	Norway	Telemedicine Symptoms control Communication User's Experience	Several	Qualitative study on digital technologies used in PC delivery at home through users' experiences.	[24]
Ohta R., Ryu Y.	2021	Japan	Communication Counseling & Decision support User's Experience	Information and communication technology (ICT)	Mame-net is an ICT system that enables the documentation of updated information about patients.	[40]
Weaver M.S., et al.	2021	USA	User's Experience	Survey Instruments	Systematic review on available survey instruments to assess patients and caregivers' perceptions of telehealth.	[63]
Allsop M.J., et al.	2022	UK	Communication	Electronic Palliative Care Co-ordination System (EPaCCS)	EPaCCs are electronic registers that enables the documentation of updated information about patients.	[41]
Brody A.A., et al.	2022	USA	Telemedicine Symptoms control Communication Education	Several	Revision of the technologies used in two clinical trials during patients' transition from the hospital to home.	[25]
Busse T.S., et al.	2022	Germany	Communication Implementation Protocol	Electronic cross-facility health record (ECHR)	ECHR are electronic registers that enables the documentation of updated information about patients and also allows its exchange among institutions.	[42]
May S., et al.	2022	Germany	Usefulness User's Experience	Several	Qualitative study on digital technologies used in PC routine through HCP's experiences.	[64]
Mo J., et al.	2022	UK	Usefulness User's Experience	Virtual Reality (VR)	Systematic review and meta-analysis on the usefulness of VR - an immersive individual three-dimensional world which might require a headset and/or remote - in PC.	[65]
Barnestein-Fonseca P., et al.	2023	Spain	Education Implementation protocol	Digital Volunteer Training Program	ITV-Pal Program - Tech-Volunteer Program in PC – to support patients and their relatives.	[59]
Cruz S., et al.	2023	Portugal	Symptoms control	Mobile Apps	Review on the application for mobile devices that support PC delivery at home.	[26]
Grimminger S., et al.	2023	Germany	Implementation protocol	AI-supported work system	PALLADiUM is a project that aims to design an AI-supported system that supports communication and collaboration in PC.	[67]
Oelschlägel L., et al.	2023	Norway	Telemedicine Symptoms control Communication User's Experience	Mobile App	Remote home care is supported by a mobile app called "Remote Home Care" (RHC) that enables remote monitoring and symptoms control.	[27]
Ott T., et al.	2023	Germany	Telemedicine Symptoms Control	Smart Sensor Technologies (SST)	Review on Smart Sensor Technologies (SST) - sensors combined with ML to evaluate data - used in PC.	[28]
Suslow A., et al.	2023	Germany	Communication Users' Experience	Information System Palliative Care (ISPC)	ISPC is a communication software that enables the documentation of updated information about patients and also allows its exchange among institutions.	[43]
Vu E., et al.	2023	Switzerland	Decision support	Machine Learning (ML)	Systematic review on ML technologies used in PC.	[7]
Antonio M.G., Veinot T.C.	2024	USA	Communication Symptoms control Users' Experience	Several	Mixed methods study on digital technologies used by chronic obstructive pulmonary disease (COPD) patients.	[29]
Griffin J.M., et al.	2024	USA	Telemedicine Communication	Teleconference	Transition to remote home care is supported by teleconsultations.	[44]
Šafran V., et al.	2024	Slovenia	Symptoms control Communication Decision support	Multilingual Framework	Multilingual Framework for Risk Assessment and Symptom Tracking (MRAST) collects, integrates, and analyzes patient-generated data. It includes an FHIR server for data storage and an automatic speech recognition (ASR) system for multiple languages.	[30]
Stanley S., et al.	2024	UK	Communication Counseling & Decision support User's Experience	Several	Mixed methods study on digital technologies to support communication in PC.	[45]
Wicki S., et al.	2024	Switzerland	Acceptance	Several	Study aiming to explore patients’ acceptance of digital technologies in PC.	[66]

**Fig. 2:**
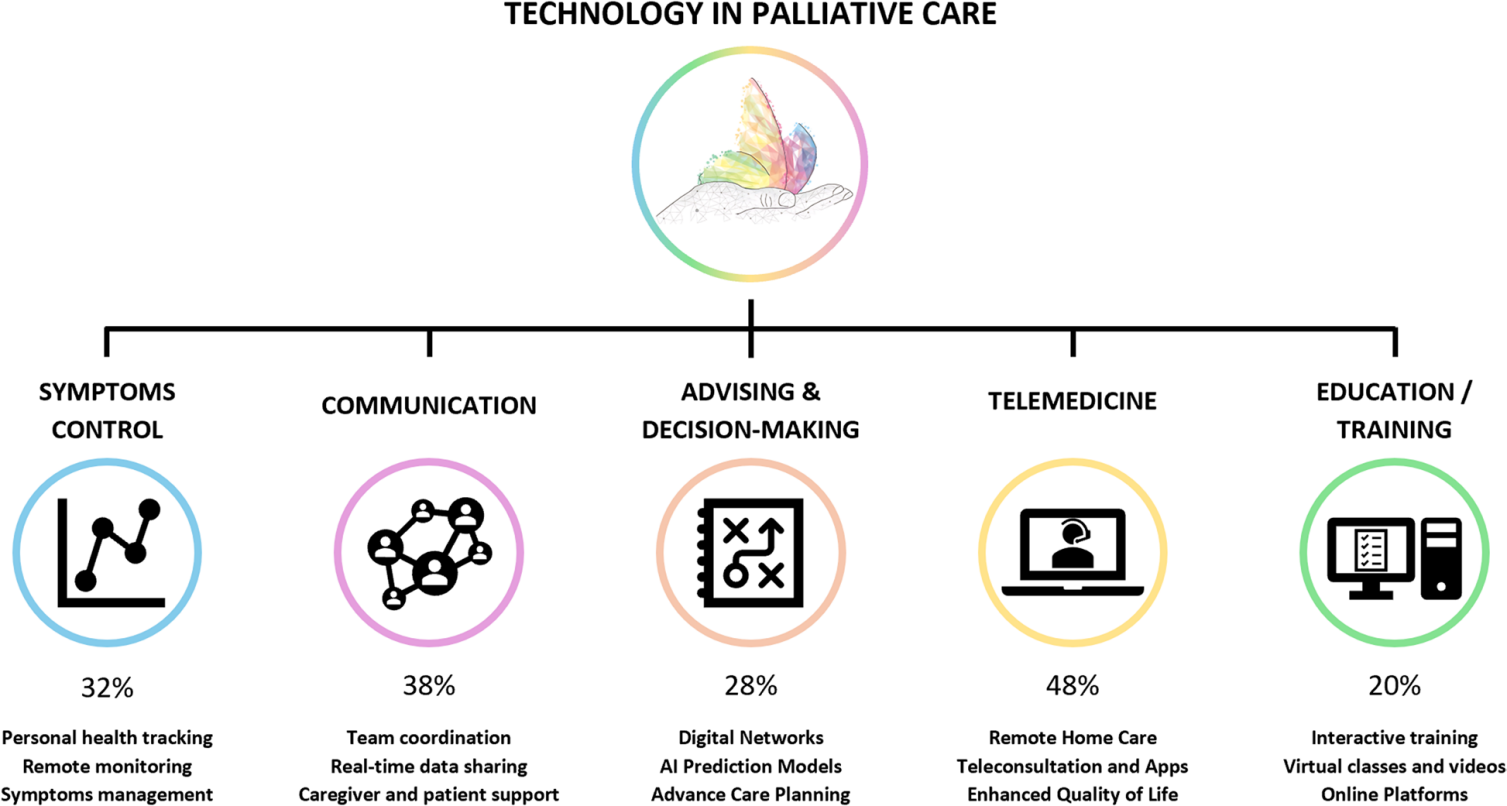
Summary of targeted technology applications in palliative care. Schematic overview of the main areas of intervention for technology in palliative care, along with the percentage representation of each area in the included articles. "Telemedicine" emerged as the most frequently addressed domain, featuring in approximately 50% of the studies, followed by "communication," cited in 40% of the records. "Symptoms control" was explored in 33% of the articles, while "advising & decision-making" appeared in 29%. The least represented area was "education/training," reported in 22% of the included studies

Throughout the next sections, we will provide more detail on the main areas of activity of the identified digital technologies in PC, as well as the main barriers to their use (Fig. 2).

### Personal health tracking – symptoms control

AI solutions have shown great potential to provide increased efficiency in the diagnosis of advanced conditions and anticipation of complications. Tools like predictive models to identify disease progression, as well as ML algorithms, may be used to monitor symptoms, stratify patients into risk categories, and predict health status [7]. Consequently, the best care plan may be tailored for each patient, thus offering timely and targeted interventions. Indeed, one of the most promising AI applications in PC is personalized advanced care planning (ACP). AI may be used to analyze a broad quantity of patient-specific data, including electronic medical records, genetic information and patient-centered research, and to identify patterns or trends that facilitate plan customization to the personal needs of each patient [12, 16, 17, 41, 42].

Likewise, several studies explored the integration of mobile health solutions and wearable devices for symptom tracking. Wearable devices and smartphone applications can track various parameters, including vital signs, physical activity, and subjective symptom ratings, with studies demonstrating high patient compliance and acceptance of continuous monitoring [19, 22]. Research indicates that certain mobile health features, such as resting heart rate and step speed, may serve as biomarkers for predicting unplanned hospital readmissions [22].

Illustrative examples where this was distinctly demonstrated are the studies by Lind & Karlsson (2004) and Lind et al. (2008), which combined mobile system with digital pen linked to an online platform, where patients recorded pain levels at home [13, 14], and Cox et al. (2011), which tested and evaluated the use of HealthHUB™, a computerized self-assessment tool for patients, alongside CareHUB™, a complementary interface for clinicians, as part of a system for monitoring in PC [16].

Overall, predictive models and mobile health solutions have consistently shown feasibility and patient acceptability, but evidence remains limited to pilot projects with small samples. Few studies have tested real-world integration into routine palliative care.

### Communication

Digital technologies are further revolutionizing communication in PC, enhancing coordination, information sharing, and support among all stakeholders. Communication emerged as a major domain for digital technologies in PC, ranging from simple yet effective tools such as messaging apps to complex collaborative platforms.

Early initiatives, such as the Keep-in-Touch (KIT) project, demonstrated feasibility for maintaining patient–family contact, while more recent studies have emphasized virtual support groups, online communities, and teleconsultation platforms to sustain peer interaction and professional collaboration [29, 33–35, 39, 46, 47, 66]. For instance, the use of centralized digital records, which ensure that patient information, preferences, and values are consistently updated and readily accessible in real-time [37]. However, studies also report challenges, including inconsistent access to ACP documentation, concerns about data confidentiality, and technical limitations [12].

The COVID-19 pandemic further accelerated the use of telephone advice lines, videoconferencing, and electronic care plans, illustrating the potential for digital communication to maintain service delivery under challenging circumstances [38, 68].

Overall, these technologies were valued for reducing isolation, enhancing coordination, and supporting shared decision-making, though concerns about confidentiality, access, and technical barriers persist.

### Healthcare provider decision support

AI has the potential to significantly impact healthcare by supporting HCPs in various aspects of their work, such as time management, communication, resource allocation, and training [51, 52]. Specifically, as previously discussed, AI technologies and predictive models can assist in diagnostics and data analytics, supporting decision-making [7]. Among the 57 studies reviewed, 12 developed or tested machine learning (ML) models to predict treatment response, mortality, or morbidity, with 5 studies reporting external validation and 2 applying models prospectively in clinical settings [7, 69–71].

These predictive models contributed to early palliative care referrals and tailored ACP by stratifying patients based on clinical trajectories and expected outcomes. Some studies reported increased confidence among HCPs when supported by decision algorithms, especially in identifying high-risk patients or prioritizing care resources efficiently. However, the strength of this evidence was generally lower, mostly based on qualitative reports or small pilot studies.

Additionally, AI-enabled communication tools and videoconferencing platforms supported shared decision-making among professionals by facilitating multidisciplinary consultations and real-time access to patient data [33, 40, 46]. These systems enhanced care coordination and reduced delays in clinical decision-making.

From the patient's perspective, education and information provided by technological tools lead to increased awareness and understanding of their care options and likely outcomes, making them more confident in their own end-of-life decision-making [48].

### Telemedicine – remote care

Consequently, the use of digital health technologies to enhance communication and monitoring has demonstrated the feasibility of remote PC delivery. Telemedicine interventions aim to improve symptom management, ensure continuity of care, and enhance communication between patients, families, and healthcare providers [36, 44, 51, 52].

Among the studies reviewed, two by Oelschlägel et al. (2021, 2023) provided detailed evaluations of a remote PC intervention using welfare technology in home settings [24, 27]. The intervention in both studies was the same: patients received a tablet and connected medical devices for real-time symptom and physiological data tracking. Simultaneously, the HCPs monitored patient data remotely and maintained regular communication with patients and caregivers via a dedicated application. The first study, based on focus groups and interviews with interdisciplinary healthcare professionals, reported a shift toward more person-centered care and increased responsiveness [24]. The 2023 study, involving patients’ perspectives, highlighted both the benefits of autonomy and communication and the burden associated with self-management of care responsibilities [27].

While these findings illustrate the feasibility and potential benefits of remote PC delivery, few additional robust studies were identified. Most evidence in this domain remains exploratory, with limited large-scale evaluations or quantitative outcome data. This highlights a gap in high-quality research assessing the effectiveness, safety, and sustainability of telemedicine approaches in PC.

### Education and training

A different aspect in which digital technology has also been proven extremely useful is training, not only for HCPs and students but also for caregivers. However, to effectively integrate AI into healthcare practices, HCPs need to develop AI literacy, including understanding data governance, statistics, and clinical process impacts [47].

Among the reviewed studies, most training interventions were based on pilot implementations or qualitative evaluations. These included the use of video tutorials, e-learning platforms, and virtual guidance tools for informal caregivers in home-based palliative care contexts [53, 56, 57]. Reported outcomes included improved caregiver confidence, enhanced understanding of care tasks, and reduced emergency service utilization.

One randomized controlled trial (RCT) by Griffin et al. assessed the impact of remote training using video consultations between the PC team and family caregivers in rural areas [44]. Although no differences in caregiver burden were noted, it was possible to observe significant improvements in general QoL and a decrease in depression symptoms.

Despite these promising findings, high-quality evidence on the effectiveness of digital training interventions in PC remains limited. Most studies are small-scale and lack long-term follow-up, highlighting the need for more rigorous evaluation.

### Barriers to implementation

Despite significant advances, several barriers continue to hinder the implementation of AI solutions in clinical routines. Even incorporating new digital tools in existing systems, such as EHR, still presents interoperability hurdles, due to data fragmentation and inconsistent standards across systems. [11, 12, 41]. Ethical issues related to data privacy and the responsible use of algorithms have also been widely discussed [7, 12, 30, 39, 45], as well as the accuracy and safety of the algorithms used. The literature points out that many AI models are developed with limited sample representation, which may reduce their applicability, algorithmic transparency, and clinician trust further complicate adoption [7]. The dependence on high-quality data to train AI algorithms is a recurrent problem. Thus, to maximize benefits, AI in PC must be trained with representative data, avoiding biases that might negatively impact vulnerable populations, and undergo rigorous external validation testing [72]. Additionally, regulatory uncertainty surrounding the development, approval, and clinical integration of AI tools poses a significant obstacle, as evolving guidelines and a lack of standardized frameworks are still evolving and often lack clear guidelines for clinical use.

Furthermore, resistance from HCPs is one of the most important bottlenecks identified for a broader adoption of AI solutions in PC [62]. This resistance mainly stems either from a lack of adequate training or the perception that AI may dehumanize care. Indeed, the integration of technologies into clinical practice without compromising ethics and humanized care remains challenging [64, 66]. PC should be profoundly centered on the patient, and introducing new AI technologies must respect human nature and feelings and preserve empathy in care. As some authors have pointed out, the balance between technology and human interaction is essential. AI should complement, not replace, the role of HCPs [52, 61, 64].

## Discussion

This scoping review of 57 studies mapped the current landscape of AI applications in PC, identifying promising areas of innovation alongside significant limitations in evidence quality and implementation readiness. In recent decades, to respond to the identified needs, new approaches for delivering modern PC based on AI technologies have emerged. The review covered a wide range of AI-enabled interventions, including predictive models for early referral, symptom monitoring systems, decision-support tools, communication platforms, and educational resources for professionals and caregivers.

Remote symptom monitoring emerged as a major focus area. Tools such as digital pens, wearable sensors, and smartphone apps allow patients to track symptoms and transmit data in real-time to clinical teams [13, 14, 16, 22]. In some cases, automatic alerts were generated when thresholds were exceeded, prompting timely interventions. While these systems improve patient engagement and continuity of care, their clinical validation remains limited.

AI-assisted screening and trigger-based models embedded in EHR represent another area of opportunity. These systems can help automate the detection of palliative needs through clinical indicators, supporting standardized referrals while optimizing healthcare resource allocation [7, 70]. However, integration into existing workflows, especially in under-resourced settings, is still a challenge.

Furthermore, predictive algorithms for treatment response, mortality, or hospitalization risk [7, 69–71] are helping identify patients who may benefit from early PC integration. These tools support stratification of patients based on clinical need and trajectory, potentially improving the timing and appropriateness of referrals.

Moreover, effective communication is key to adequate planning and provision of PC in both hospital and community/home settings [73]. New technologies and AI may assist in the establishment of communication platforms to ease this process, promoting practice models of integrated PC in contrast to less adequate solo practice models [74]. Several included studies examined how AI and digital platforms improve information flow among interdisciplinary teams, patients, and families. Tools such as Electronic Palliative Care Co-ordination System (EPaCCS) and Information System Palliative Care (ISPC) [37, 43] facilitate shared documentation of patient preferences, care plans, and clinical updates. These innovations promote collaborative practice and improve care transitions. Additionally, the KIT project and CovPall survey [35, 38] showed how digital tools reduced isolation and supported remote care delivery, respectively.

Interestingly, while the COVID-19 pandemic acted as a catalyst for the adoption of digital health tools, the long-term sustainability of these interventions remains uncertain. Many of the rapid implementations lacked standardized evaluation frameworks, stable funding, or integration into health systems. Post-pandemic, maintaining momentum requires embedding digital innovations into routine care pathways, ensuring interoperability with EHRs, and developing clear regulatory and reimbursement policies. Without such systemic support, there is a risk that some solutions may regress to pilot status rather than evolve into sustainable, scalable practices [45, 58].

That being said, developing AI literacy among clinicians is essential. Digital education resources support capacity-building for both professionals and informal caregivers. While several studies used video content, e-learning, or virtual coaching to deliver training [53, 56, 57], only one RCT [44] evaluated outcomes such as caregiver quality of life and mental health. Most evidence remains qualitative or pilot-level, limiting generalizability. In addition, studies highlighted the need for training in data governance, bias awareness, and ethical considerations [47, 52] especially to overcome resistance rooted in fears of depersonalization or reduced professional autonomy.

Nevertheless, numerous barriers hinder the implementation of AI in PC. Technical barriers include a lack of interoperability with EHRs, insufficient data quality, and algorithmic bias due to non-representative training datasets and regulatory uncertainty [11, 12, 30, 41]. For instance, recent literature underscores the risks of algorithmic bias and exclusionary systems, particularly in underrepresented populations [75].

On the other hand, human and organizational barriers such as resistance from HCPs, limited training, and concerns about ethical integrity are also common [51, 61, 65]. Equity, diversity, and inclusion (EDI) considerations are critical when evaluating the integration of AI in PC. Most included studies originated from high-income countries, often with homogeneous populations, which raises concerns about the representativeness of datasets used to train AI models. Biases in these datasets can reinforce disparities, disproportionately disadvantaging vulnerable groups such as ethnic minorities, rural communities, or patients with limited digital literacy. Cultural sensitivity is equally relevant, as perceptions of technology, trust in digital tools, and end-of-life preferences vary widely across sociocultural contexts. Future research must ensure inclusive design, participatory approaches, and culturally adapted implementation strategies to avoid widening existing inequities in PC access and quality [76].

### Study limitations

Additionally, our study presents some limitations that must be acknowledged. First, restricting the search to two databases (PubMed and Scopus) and to English-language publications may have limited the comprehensiveness of the evidence captured. While these databases cover the majority of biomedical and health sciences journals, relevant studies published in other languages or indexed in alternative databases (e.g., Web of Science, IEEE Xplore) may not have been retrieved. Consequently, the findings may underrepresent perspectives from non-English-speaking countries and certain technological domains.

Second, the search strategy combined the terms “palliative care” and “technology”, with no additional filters applied. This approach, albeit narrow, was chosen to ensure feasibility and specificity, focusing on studies explicitly addressing the intersection of digital technologies and PC. Nevertheless, we acknowledge that this strategy may have limited sensitivity, as terms such as “machine learning”, “decision support”, or “predictive modeling” were not included as primary search terms, potentially underrepresenting certain technological applications. However, reference lists of retrieved records were hand-searched to identify additional relevant studies that may not have been captured through the initial query.

Third, grey literature was not included. Although this decision was made to ensure methodological rigor, transparency, and feasibility of the review process, it may have excluded valuable insights, particularly in the field of digital health technologies, where many innovations are initially reported in conference proceedings, technical reports, or project documentation. By focusing on peer-reviewed publications, we prioritized studies with formal evaluation and quality assurance processes. Nevertheless, we recognize that omitting grey literature may have led to the underrepresentation of early-stage implementations and pilot projects, and future reviews should consider including this type of evidence to provide a more comprehensive picture.

Another notable concern across the studies reviewed is the overall quality of evidence. Most were feasibility studies or descriptive evaluations with small sample sizes and short durations. Only a minority used robust study designs, and external validation of AI models was rare. Withal, the predominance of exploratory or pilot studies, heterogeneity of interventions, outcomes, and populations restricts the comparability and generalizability of conclusions to broader PC populations.

### Future perspectives

Ultimately, from the retrieved literature, we could identify several suggested directions for future research in AI and PC. First, interdisciplinary collaboration among technology developers, HCPs, and patients is key for the development of humanized and culturally sensitive tools. Second, there is a need to create supportive policies that encourage education and training of HCPs to increase acceptance and trust in AI. One of the included studies highlights that the involvement of community networks and the strengthening of social support are essential for the success of these initiatives [29]. Third, several protocol and implementation studies offer examples of best practices for achieving success in this area, as well as suggestions for future improvements. One of such study focused on technological resources that enhance volunteer interaction and communication within the community. Volunteers with training in new technologies will be able to help patients with daily practical tasks. A training program for volunteers will be evaluated and a program of follow-up using technological devices will be implemented to support patients facing a life-threatening illness and their relatives [59]. As another example, the project PALLADiUM will explore the potential and limitations of digitally supported communication and collaborative solutions, in which AI-based approaches are suitable for making structured and unstructured data in PC more usable for communication and collaboration processes. The work system serves as a blueprint for a technical prototype aiming to improve communication and collaboration in the multi-professional team of the PC unit [67].

## Conclusions

This mapping study highlights the transformative potential of AI-based technologies in PC while emphasizing the need for thoughtful, patient-centered implementation. As the field rapidly evolves, we stand at a pivotal moment to develop solutions that not only enhance care quality but also uphold patients' dignity and autonomy. Future research must address technical, ethical and organizational challenges, ensuring that AI becomes a tool that not just advances but truly humanizes PC, making personalized care more accessible and effective.

## Supplementary Information

Supplementary Material 1.

## Data Availability

Not applicable.

## Abbreviations

ACP: Advanced Care Planning
AI: Artificial Intelligence
CDSS: Clinical Decision Support Systems
EDI: Equity, diversity, and inclusion
EHR: Electronic Health Record
EPaCCS: Electronic Palliative Care Co-ordination System
HCP: Health Care Professionals
ISPC: Information System Palliative Care
KIT: Keep-in-Touch
ML: Machine Learning
PC: Palliative Care
QoL: Quality-of-Life
RCT: Randomized Controlled Trial
WHO: World Health Organization
