# Supplementary material for: Mapping study on AI-based technologies in palliative care – a scoping study
Source: BMC Palliat Care. 2025 Oct 28;24:274. doi: 10.1186/s12904-025-01909-w (PMC12570659; doi:10.1186/s12904-025-01909-w)
Supplement: Supplementary file 1 — Supplementary Material 1. [file 12904_2025_1909_MOESM1_ESM.pdf]

Electronic Search Strategy

Query for Pubmed

(((“palliative care” [MeSH Terms]) OR (“palliative medicine” [MeSH Terms])) AND (“technology” [MeSH Terms]))

Query for Scopus

INDEXTERMS((("palliative care" OR "palliative medicine") AND ("technology")))
